# Parallel Imaging of 3D Surface Profile with Space-Division Multiplexing

**DOI:** 10.3390/s16010129

**Published:** 2016-01-21

**Authors:** Hyung Seok Lee, Soon-Woo Cho, Gyeong Hun Kim, Myung Yung Jeong, Young Jae Won, Chang-Seok Kim

**Affiliations:** 1 Department of Cogno-Mechatronics Engineering, Pusan National University, Busan 609-735, Korea; leehsx@pusan.ac.kr (H.S.L.); swcho6234@pusan.ac.kr (S.-W.C.); apfield@pusan.ac.kr (G.H.K.); myjeong@pusan.ac.kr (M.Y.J.); 2 Medical Device Development Center, Osong Medical Innovation Foundation, Cheongju, Chungbuk 361-951, Korea

**Keywords:** laser and laser optics, swept source, optical frequency domain imaging, three-dimensional imaging, interferometry

## Abstract

We have developed a modified optical frequency domain imaging (OFDI) system that performs parallel imaging of three-dimensional (3D) surface profiles by using the space division multiplexing (SDM) method with dual-area swept sourced beams. We have also demonstrated that 3D surface information for two different areas could be well obtained in a same time with only one camera by our method. In this study, double field of views (FOVs) of 11.16 mm × 5.92 mm were achieved within 0.5 s. Height range for each FOV was 460 µm and axial and transverse resolutions were 3.6 and 5.52 µm, respectively.

## 1. Introduction

Concomitant with the rapid growth of the precision components industry, surface profile measurement has become increasingly important in industrial inspection processes and many nondestructive three-dimensional (3D) surface profiling techniques [1,2,3,4] have been developed for product quality control and process efficiency. Further, as demand for cost-effective, noncontact, high-speed, high-accuracy 3D surface profiling technology is on the rise, various optical approaches to 3D surface profile imaging have been widely suggested [5,6,7,8,9]. Axial scanning methods with mechanical displacement change are a common set of approaches used to record the amplitude variance on a sample surface in an optical interferometer [7,8,9]. However, approaches that employ mechanical movement are susceptible to hysteresis of the mechanical translation, which makes them unsuitable for imaging systems. 

Alternatives that employ a tunable laser source to build non-mechanical movement optical imaging systems have been extensively studied [10,11,12,13]. In particular, optical frequency domain imaging (OFDI) technology, also known as swept source optical coherence tomography (SS-OCT), has been intensively studied as a means of avoiding mechanical movement in axial scanning by using alternative wavelength scanning of the swept source. The principle of OFDI is based on a tunable wavelength and an optical interferometer using it. The output of the light source is split into a reference arm and a sample arm which guides the light reflected from the sample. The interference between the reference and sample arm light is detected with a photo detector while the wavelength of the light source is swept and the path lengths of the reference and sample arm are held constant [11,12,13]. Compared to a traditional optical imaging system using a time-domain process with a white-light source, the OFDI image thus obtained also exhibits merit such as enhanced signal-to-noise ratio (SNR) and rapid imaging acquisition [14,15,16]. As the swept source changes its center wavelength of light to propagate through an optical interferometer, the OFDI system acquires spectrally resolved interference signals to process the displacement information by frequency domain analysis [17]. Because this displacement information along a light path can only be obtained by the wavelength-encoding of the swept source, we are able to successfully image a 3D surface profile in the area dimension using multiple simultaneous light paths when the area beam is illuminated in the OFDI system without any mechanical movement [14,18]. The field of view (FOV) of this area-beam type OFDI system is simply fixed by the dimensions of a single-area beam and a single imaging camera [14].

Recently, as imaging depth range can easily be extended in the conventional point-beam interferometer system, a space division multiplexing (SDM) method was suggested to obtain multiple displacement information from multiple point beams using a single detector in the same time [19,20]. Increase of the 3D surface profiling speed becomes very important in the real-time inspection of industrial fabrication fields such as solid bumps on printed circuit board (PCB), light emitting diode (LED) package, image sensor module (ISM), *etc.* Since our area-beam type OFDI system is also based on an interferometer, the SDM method can be newly applied to extend from point beam to area beam. It means that simultaneous measurement of multiple-area 3D surfaces can be proved in a new SDM-OFDI system with multiple-area swept source beams.

In this study, for the first time, we experimentally demonstrated a SDM-OFDI system with dual-area swept source beams to obtain 3D surface information for two different areas at the same time by using a single camera. We prove our method by measuring 3D surface profiles of two different areas in a coin.

## 2. Space Division Multiplexing with Dual-Point Swept Source Beams

**Figure 1:**
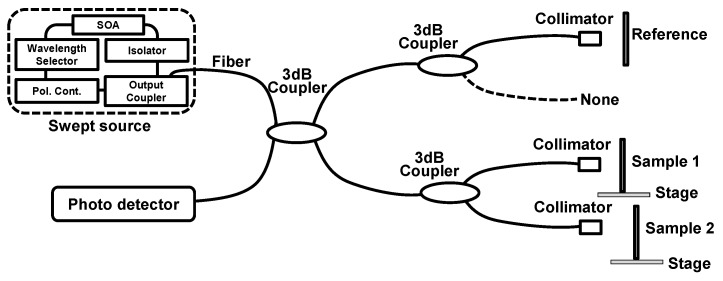
SDM-OFDI system with dual-point swept source beams.

To see the characteristics and capability of our method, we first demonstrated the SDM-OFDI system with dual-point swept source beams as shown in Figure 1. In this experiment, the optical interferometer consists of a swept source, three 3 dB couplers, three collimators, one reference arm, two sample arms, and one photodetector. Two stages are located in both sample arms to change the displacement of the two samples on them. The swept source with a center wavelength of 800 nm was used as a light source. This swept source consisted of a fiber-coupled semiconductor optical amplifier (SOA) module, a wavelength selector, optical isolators, an inline polarization controller (PC), and an output coupler. The sweeping range and maximum output power of the light source were 50 nm and ~20 mW, respectively. The linewidth of the swept source was measured to be less than 0.05 nm; thus, it is a long coherence length greater than can be expected. The swept source laser beam went through a 3 dB coupler and divided into reference and sample arms to realize the interferometer. In the reference and sample arms, each beam was also injected into extra 3 dB couplers to demonstrate SDM method with dual-point swept source beams. In the sample arm, each beam after the extra 3 dB coupler went to each sample after passing through a collimator. In the reference arm, one of two beams after the extra 3 dB coupler was used as a reference. The used optical fiber in the SDM-OFDI system was Corning HI780 to ensure single transverse mode operation.

**Figure 2:**
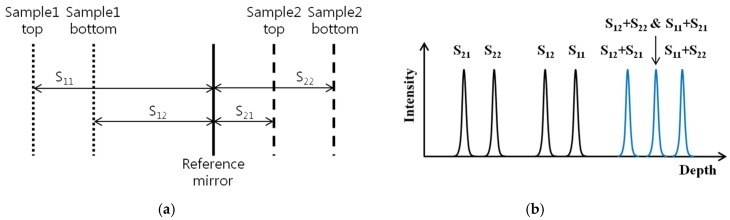
(**a**) Distance between sample1, sample2 and reference mirror; (**b**) Parallel measurement of two 3D surface profiles.

In this system, there were three parts to interfere: (1) reference and sample 1; (2) reference and sample 2; (3) sample 1 and sample 2 such that the distance between them must be carefully considered to distinguish them clearly by our method. By moving the position of each stage, the optical path length of sample 1 and sample 2 can be easily controlled to divide the total coherence length of light source into two depth information regions. The case for the placement of samples and the reference mirror is shown in Figure 2. Here, S11 and S21 represent the distance between the reference mirror and the top of each sample, respectively. S12 and S22 represent the distance between the reference mirror and the bottom of each sample, respectively. In this case, the height information of two samples can be well differentiated with the condition of S21 < S22 < S12 < S11 < S12 + S21.

To demonstrate the parallel acquisition of 3D information for two samples, cover glass with a thickness of 140 µm was used in both sample arms. The wavelength-encoded optical interference signal from the two samples and the reference mirror was detected by a photo detector and converted into the electric signal. Then, the depth-encoded signal was obtained from the wavelength-encoded signal with the Discrete Fourier Transform (DFT) method [21].

**Figure 3:**
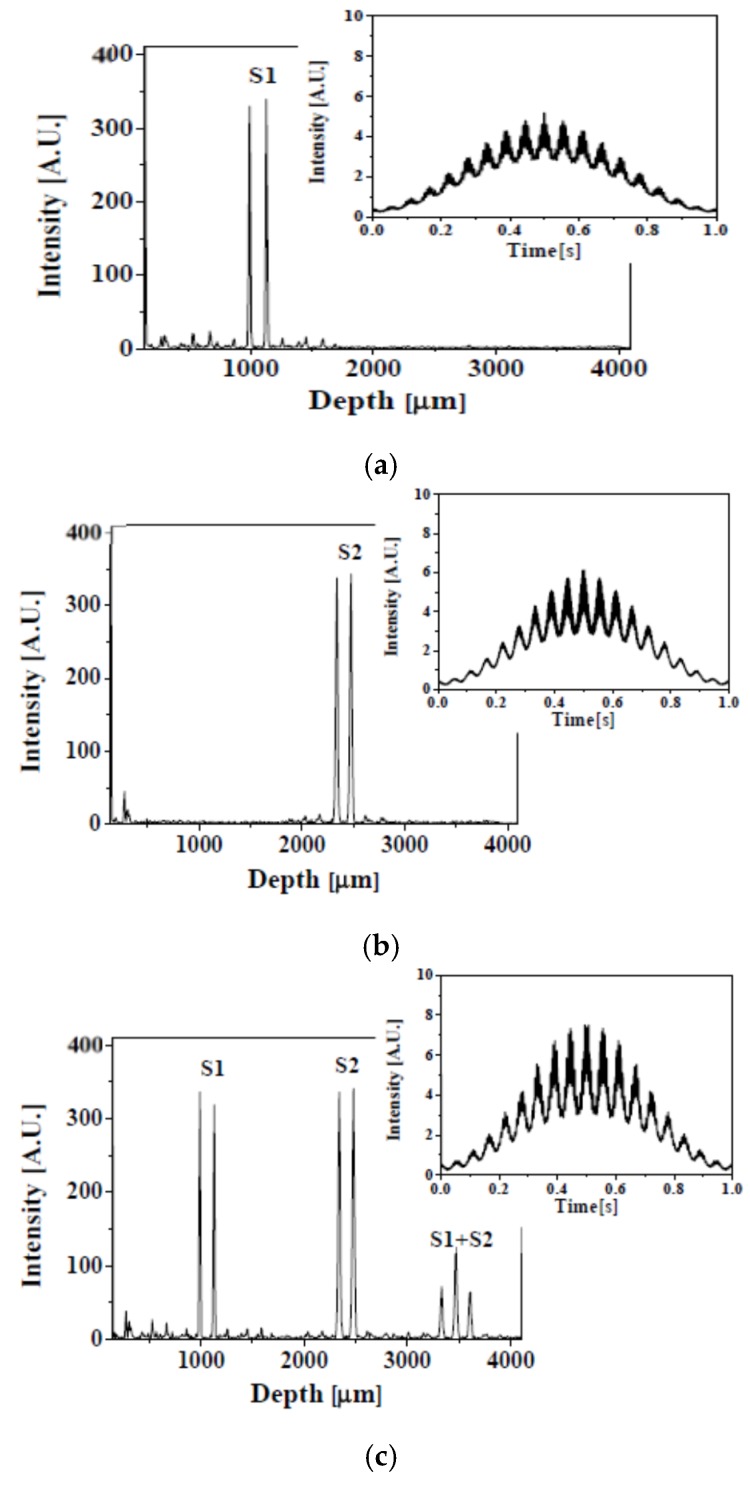
(**a**) Depth-encoded signal with only sample1; (**b**) Depth-encoded signal with only sample 2; (**c**) Depth-encoded signal with both sample1 and sample 2.

Figure 3 shows the depth-encoded signal acquired by the SDM-OFDI system with dual-point swept source beams. In this experiment, interferometric information was acquired from one cycle scanning of the swept source, as shown in the insets of Figure 3, and it was converted to the depth range information along about 4000 µm. Sample 1 was placed about 1000 µm after the reference mirror and sample 2 was placed about 2400 µm before the reference mirror. The depth information due to interference between both samples appeared after 3200 µm so that the depth information for two samples can be well distinguished by each one with the SMD-OFDI system with dual-point swept source beams. Though the single photo detector receives a dual-frequency interferogram, each individual frequency contrast is not degraded because the dual frequency fringes are clearly separated along the depth scale during the DFT process, as represented in Figure 3. If the coherence length of the used swept source is increased, the available depth range for two samples is proportionally increased or the number of samples to measure simultaneously is also increased [18].

## 3. Space Division Multiplexing with Dual-Area Swept Source Beams

The camera-based 3D surface measurement OFDI system with a swept sourced interferometer has many advantages such as simple design, free for depth scan, and fast measurement time due to parallel data processing with a computer-unified device architecture (CUDA). Moreover, multiple FOVs for 3D surface measurement are obtainable despite only single camera-based system when the SDM method is applied with dual-area swept source beams.

**Figure 4:**
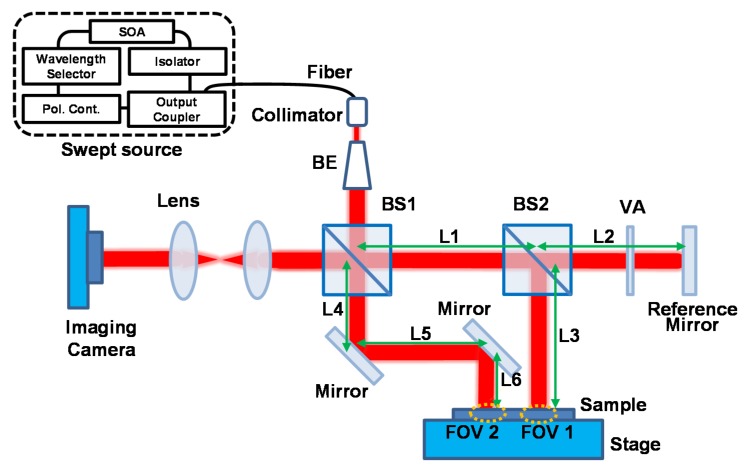
SDM-OFDI system with dual-area swept source beams.

The experimental setup for the SDM-OFDI system with dual-area beam illumination is represented in Figure 4. The used light source is the same as the one mentioned in Section 2. It was collimated and resized as larger than 10 mm after passing through a collimator and beam-expander. Dual-area beam illumination was realized by using two beam splitters (BS1 and BS2). Most of the interference signals in the FOV1 area are simultaneously induced by the optical path length difference between L1 + L2 and L1 + L3. Similarly, the sample information of FOV2 comes from that between L1 + L2 and L4 + L5 + L6. In order to distinguish the area distance between FOV1 and FOV2 on the same plane, the above three kinds of distances should be adjusted suitably to divide the total coherence length of the light source into two height information regions. The 2D interference signals with respect to different wavelengths were passing through two convex lenses (f = 200 mm) and were acquired in order by a Si-type charge-coupled device (CCD) with 8-bit resolution. The pixel size of the CCD was 5.5 µm × 5.5 µm and the CCD consisted of 2040 × 1088 pixels such that the sensor size of the CCD was 11.22 mm × 5.98 mm. In this experiment, 170 frames for 2D interference signals with respect to different wavelengths were used to extract depth-resolved 3D surface information. Since a frame rate of CCD was 340 frames/s, the total acquisition time was 0.5 s. To protect the signal saturation and enhance the interference efficiency, the output power of the light source and the attenuation of the variable attenuator in the reference arm can be optimally controlled.

**Figure 5:**
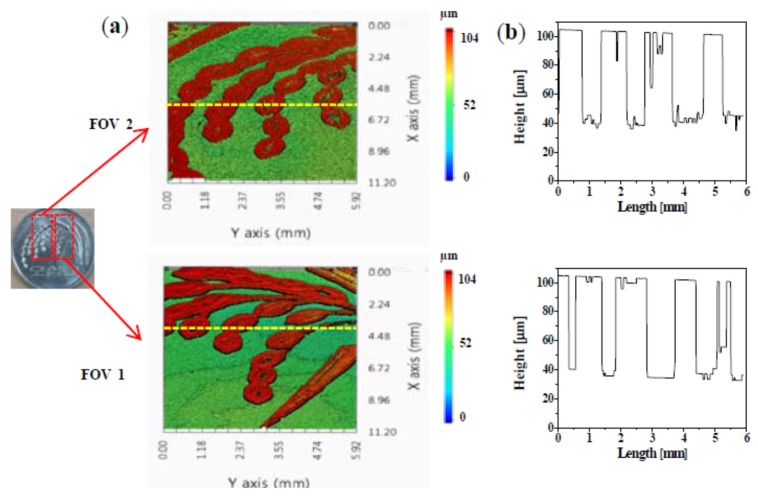
(**a**) Two different 3D surface profiles of a coin measured by SDM-OFDI system with dual-area swept source beams; (**b**) Cross-sectional profile along the dash line of each 3D surface profile.

Figure 5a shows two different 3D surface profiles of a coin measured by the SDM-OFDI system at the same time with dual-area swept source beams, and their cross-sectional profile along the dash line of each 3D surface profile is also represented in Figure 5b. Compared with the conventional camera-based 3D surface measurement system, the SDM-applied OFDI system with dual-area swept source beams can measure multiple 3D surface profiles of different areas at the same time. It can provide the effect for increment of FOVs multiple times despite being a single camera-based optical system. We obtained two FOVs of 11.16 mm × 5.92 mm with only a single camera-based system and the acquisition time was 0.5 s. To obtain the axial resolution of the depth-encoded signal in the imaging process, we measured the cover glass with a thickness of 140 μm using the proposed SDM-OFDI system and found out the digitized resolution of about 3.6 μm in the condition of a 512 data sampling number for DFT. The validation of the result also confirmed that the average height difference on the coin surface sample (200 points for each top and bottom region) shows a similar value of about 64.8 and 65.39 µm for both FOV 1 and 2, respectively.

For the comparison, the other profiling method of white light scanning interferometry (WSI; NV-2400, Nanosystems, Inc., Daejeon, Korea) was also applied to the same coin sample with a much smaller FOV of 466.6 μm × 622.2 μm (×10 objective lens) to obtain the similar average height of 68.6 μm.

In case of the measurement of transverse resolution, we applied this system with the standard U.S. Air Force resolution target. The measured transverse resolution was 5.52 µm, which exhibits reasonable agreement with the theoretical calculation and with the CCD dimension and pixel size. Since we can expect a single height range of 920 µm from the 256 depth numbers multiplied by a depth pixel of 3.6 µm, the height range for each sample 1 and sample 2 was about 460 µm by dividing for two separate height ranges. Since these two FOV images divide the total coherence length of the light source into two height information regions, the limit of the height measurement is reduced to half of a single FOV’s. Thus, to keep a similar height measurement region, the longer coherence light source will be preferred. To remove the spiky noise in the data which comes physically from the cross-talk between CCD pixels, an additional software process is applied using the median filter with a size of 10 × 10 pixels in the 3D surface profile image. As we increase the size of the median filter, we can reduce the spiky noise effectively but there will be a trade-off relation between the smoothing effect and measurement accuracy.

## 4. Conclusions

In this research, we proposed and experimentally demonstrated a modified OFDI system based on the SDM method to achieve a parallel imaging of 3D surface profiles. The SDM-OFDI system with dual-area swept source beams makes it possible to measure 3D surface profiles for multiple FOVs simultaneously, despite being a single camera-based system. The SDM-OFDI method with dual-area swept source beams can be powerfully applied to industrial fields, especially 3D inspection equipment, because it has the many advantages of simultaneous multiple area inspection and accurate measurement due to free for depth scanning. By using a single camera, we have acquired 3D surface information for two areas of a coin at the same time within 0.5 s. The height range for each sample was about 460 µm and the axial resolution was 3.6 µm.
